# A Transcriptomic Dataset of Embryonic Murine Telencephalon

**DOI:** 10.1038/s41597-024-03421-x

**Published:** 2024-06-05

**Authors:** Shohei Ochi, Shyu Manabe, Takako Kikkawa, Sara Ebrahimiazar, Ryuichi Kimura, Kaichi Yoshizaki, Noriko Osumi

**Affiliations:** 1 Department of Developmental Neuroscience, Tohoku University Graduate School of Medicine, Sendai, 980-8575 Japan; 2 Institute of Resource Development and Analysis, Kumamoto University, Kumamoto, 860-0811 Japan; 3 Kobe University Graduate School of Medicine, Department of Future Medical Sciences, Division of Integrated Analysis of Bioresource and Health Care, Kobe, 650-0047 Japan; 4 Kobe University Hospital, Bioresource Center, Kobe, 650-0047 Japan

**Keywords:** Developmental neurogenesis, Developmental neurogenesis

## Abstract

Sex bias is known in the prevalence/pathology of neurodevelopmental disorders. Sex-dependent differences of the certain brain areas are known to emerge perinatally through the exposure to sex hormones, while gene expression patterns in the rodent embryonic brain does not seem to be completely the same between male and female. To investigate potential sex differences in gene expression and cortical organization during the embryonic period in mice, we conducted a comprehensive analysis of gene expression for the telencephalon at embryonic day (E) 11.5 (a peak of neural stem cell expansion) and E14.5 (a peak of neurogenesis) using bulk RNA-seq data. As a result, our data showed the existence of notable sex differences in gene expression patterns not obviously at E11.5, but clearly at E14.5 when neurogenesis has become its peak. These data can be useful for exploring potential contribution of genes exhibiting sex differences to the divergence in brain development. Additionally, our data underscore the significance of studying the embryonic period to gain a deeper understanding of sex differences in brain development.

## Background & Summary

Sexual reproduction is a highly successful strategy for the survival of vertebrate species, allowing them to adopt to environmental changes. One intriguing aspect of sexual dimorphism is the presence of differences between males and females in various organs, including the brain. Studies have consistently revealed that males generally possess a larger brain volume, whereas females exhibit a relatively larger ratio of gray-to-white matter^1–3^. Additionally, specific regions of the cerebral cortex have been found to be thicker in females, further highlighting the existence of sexual dimorphism^1,3–5^. Rodents, as a valuable model system, have played a crucial role in investigating sexually dimorphic brain regions associated with sexual behavior, such as the bed nucleus of the stria terminalis, the medial nucleus of the amygdala, and the medial preoptic nucleus of the hypothalamus^6,7^. Despite these advances in identifying genetic and molecular mechanisms contributing these sex differences in brain structures remains incomplete.

Autism spectrum disorder (ASD) represents a prominent neurodevelopmental disorder (NDD) characterized by its distinct features including communication deficits and repetitive behaviors as well as other comorbid symptoms. There are a few studies reporting the presence of subtle abnormalities in brain architectures associated with ASD^8,9^. Interestingly, cognitive and social behaviors have been shown to exhibit sex differences^10–12^. Moreover, it has been well-documented that ASD is more prevalent among boys, while major depressive disorder exhibits a higher incidence in girls^13,14^. Therefore, it is plausible that the minor differences in brain structure contributing to the development of ASD may also be influenced by sex differences. Thus, it becomes crucial to consider the role of sex differences in brain development when examining the broader context of NDDs, enabling a more comprehensive understanding of these conditions and their underlying mechanisms.

**Fig. 1:**
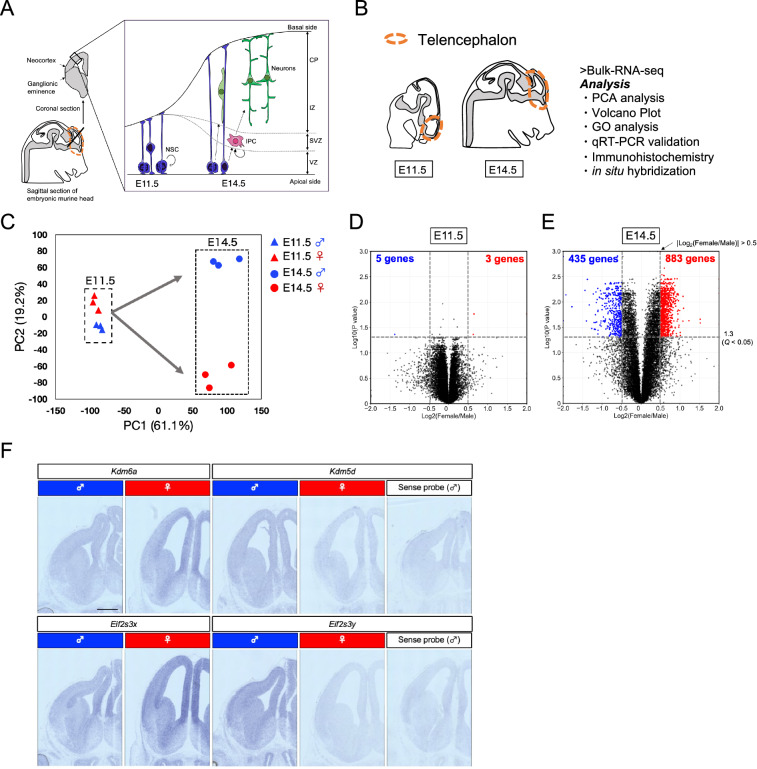
Differentially expressed genes in the embryonic telencephalon of male and female mice at E11.5 and E14.5. (**A**) During the early stages of cortical development, neural stem cells (NSCs, depicted in blue) located in the ventricular zone (VZ) undergo symmetric divisions to increase their population. Subsequently, these NSCs undergo asymmetric divisions, giving rise to intermediate progenitor cells (IPC, depicted in red) which, in turn, generate neurons (depicted in green). Over time, these neurons self-organize into distinct layers. In mouse cortical development, the embryonic stage E11.5 is characterized by active proliferation of NSCs, while E14.5 marks a period of robust neuron production. (**B**) Outlines the experimental workflow (see “Method” session in detail). (**C**) Principal Component Analysis (PCA) on RNA-seq data obtained from the embryonic telencephalon of male and female mice at E11.5 and E14.5 (n = 3). At E11.5, the gene expression profiles of individual telencephalons are similar between males and females (depicted in blue and red triangles, respectively). However, at E14.5, significant differences are observed in the gene expression profiles between male and female telencephalons (depicted in bule and red circles). (**D,E**) Volcano plots showing the differentially expressed genes (DEGs) in the embryonic telencephalon of mice at E11.5 (D) and E14.5 (**E**). The horizontal axis represents the expression ratio (log_2_(female expression level/male expression level)), while the vertical axis represents the *q*-value. The threshold is set at *q* < 0.05 and |Log_2_(female expression level/male expression level) | > 0.5, and the number of genes meeting these criteria is indicated above each plot. (**D**) At E11.5, a smaller number of DEGs are observed between males and females. (**E**) At E14.5, the number of DEGs in female was 883, compared to 435 in males. This indicates an increase in the number of these DEGs at E14.5 compared to E11.5. Furthermore, the female telencephalon showed twice the number of highly expressed genes as the male telencephalon at E14.5. A total of 13,897 and 14,173 genes are expressed at E11.5 and E14.5, respectively. The *q*-value represents the adjusted *p*-value obtained from the Student’s *t*-test with multiple comparisons correction using the Benjamini-Hochberg methods. (**F**) *In situ* hybridization of *Kdm6a* and *Eif2s3x* (female dominant expression) as well as *Kdm5d* and *Eif2s3y* (male dominant expression) in the male and female telencephalon. *Kdm6a* and *Eif2s3x* exhibited stronger signal in female samples, while *Kdm5d* and *Eif2s3y* exhibited signal only in male samples, showing trends in consistent with RNA-seq data. Scale bar: 100 µm.

The embryonic period plays a pivotal role in brain development, as most neurons are generated during this critical phase, while their subsequent positioning occurs over the postnatal period^15–17^. The vertebrate central nervous system originates from the neural plate, an ectodermal tissue that undergoes a transformative process, folding and rolling up to form the neural tube. In the mouse, the rostral portion of the neural tube expands, giving rise to two bulges and forming the telencephalic vesicles by embryonic day (E) 10.5. Initially, the vesicle consists solely of neural stem cells (NSCs), and their population expands through active cell division between E10.5 and E11.5. Around E11.5, NSCs begin to generate neurons, which migrate to the basal side of the telencephalon to establish the layered structure of the cerebral cortex^18^ (Fig. 1A). Neurogenesis, i.e., production of neurons, reaches its peak activity at E14.5 and gradually decreases by E17.5, while the migration of neurons to their appropriate positions continues^19^ (Fig. 1A). The maturation of adult brain organization is completed around postnatal day (P) 10^18,20^.

Traditional beliefs have long suggested that the brain follows a default pathway towards developing a female phenotype, and it undergoes masculinization through exposure to male sex hormones, specifically testosterone, during the perinatal stage (around E18.5 in mice)^21,22^. Among the brain regions that exhibit sexual dimorphism, the hypothalamus has garnered significant attention due to its association with reproductive behavior. Recent groundbreaking studies have shed light on the role of a specific gene, *Ptf1a*, expressed in the embryonic hypothalamus before the surge of sex hormones, as evidenced by the loss of reproductive behavior observed in *Ptf1a* knockout mice following sexual maturity^23,24^. However, it is important to note that these investigations have primarily focused on sexual differentiation in the hypothalamus, leaving open questions regarding the existence of similar genetic mechanisms in other brain regions, such as the cerebral cortex, which is responsible for higher brain functions. Further exploration in these areas is necessary to comprehensively understand the genetic mechanisms underlying sexual differentiation in the brain.

Previous studies have investigated gene expression profiles in the mammalian brain, specifically in male and female mouse whole brain or whole heads, prior to the surge of sex hormones at E10.5 and E11.5^25,26^. These studies have identified a limited number of candidate genes on the sex chromosomes that exhibit sex difference in the expression. Additionally, quantitative polymerase chain reaction (PCR) analysis of the whole mouse brain at E13.5 has shown the specific expression of six genes on the Y chromosome in males, while their homologous genes on the X chromosome display robust expression in females^27^. Despite these findings, the precise involvement of these genes in the sexual differentiation of the brain remains unclear.

Based on the aforementioned background, the current study aimed to conduct a comprehensive analysis of gene expression analysis in the cortical primordium of wild-type mice at E11.5 and E14.5. The objective was to identify differences in gene expression profiles between males and females prior to the surge of sex hormones. To investigate potential sex differences in gene expression prior to the sex hormone surge during mouse development, we conducted a comprehensive gene expression analysis using bulk RNA-seq data of the developing telencephalons at two crucial time points: E11.5 (characterized by NSC proliferation) and E14.5 (marked by massive neurogenesis) (Fig. 1A,B)^28,29^. To relate the transcriptomic differences, we validated expression of the genes of interest by *in situ* hybridization and quantitative real-time polymerase chain reaction (qRT-PCR). We also analyzed cytoarchitecture of the embryonic cortex at E11.5, E14.5 and E17.5.

## Methods

### Experimental animals

Wild-type C57BL/6 J mice, bred and housed at the Experimental Animal Facility of the Tohoku University Graduate School of Medicine, were used for the experiments in this study. Female mice were mated with male mice, and embryos at E11.5, E14.5, and E17.5 were analyzed, with the day of vaginal plug confirmation considered as embryonic day 0.5. The mice were purchased from CLEA Japan Inc. All animal experiments conducted in this study were performed in accordance with the “Regulations for Animal Experiments at Tohoku University” and complied with ARRIVE guidelines. The study was approved by the Specialist Animal Experimentation Environment and Safety Committee of Tohoku University (2020 Medical Research and Development −109-10). The animals were treated according to the National Institutes of Health guidance for the care and use of laboratory animals.

### Sex determination

The sex of mouse embryos was determined using a combination of genomic PCR and morphological observation of gonadal primordia. For E14.5 embryos, after sample acquisition, the embryos were dissected, and the gonadal primordia located on the dorsal surface of the hind limb were isolated to determine the sex based on morphological differences, following a previously reported method^30^. The sex of the samples at the other developmental stages were determined only by genomic PCR. Tails from embryos were isolated at the time of sample acquisition and added to 50 µL of tissue lysate (1× Mg^2+^-free GoTaq buffer (Promega), 0.8 mg/mL Proteinase K, 1% NP40), then incubated overnight at 57 °C on a hot plate. Proteinase K was inactivated by heating the lysate at 95 °C for 15 minutes using an incubator. The lysate was then centrifuged at 15,000 rpm at 4 °C for 15 minutes, and the supernatant was collected as the genomic extract for PCR. The following PCR conditions were used: 1 μL of the supernatant was added to the GoTaq PCR reaction mixture (1 × Mg^2+^-free PCR buffer, 0.75 mM MgCl2, 0.1 mM dNTPs, 0.5 μM *Sry* forward primer: 5′-tgaatgcatttatggtgtgtc-3′, 0.5 μM *Sry* reverse primer: 5′-aatctctgtgcctcctggaa-3′, 1 unit of Taq polymerase). The PCR amplification was performed as follows: 95 °C for 2 minutes, followed by 30 cycles of 95 °C for 30 seconds, 55 °C for 30 seconds, and 72 °C for 1 minute, with a final extension at 72 °C for 2 minutes.

### Library preparation and RNA sequencing

The whole telencephalon, including the primordia of the cerebral cortex and the basal ganglia, was dissected from E11.5 and E14.5 WT C57BL/6 J mouse embryos during the light phase of the day. To minimize potential developmental variations between male and female samples, an equal proportion of male and female samples from the same littermates was selected (Supplementary Table S3). For precise dissection of the telencephalon, fine forceps (REGINE #5) and the microscissors (Leprex, LMB-50-7) were utilized^31^. At E11.5, the eye primordium was removed first to prevent contamination of the brain sample with the eye tissue. The border between the telencephalon and diencephalon was cut using fine forceps, and only the telencephalon was collected. Any excess tissue along the border was also excised to clearly expose the lateral ventricles^32^. At E14.5, the skull tissue was carefully removed from the entire brain, and the whole brain was dissected from the head of the embryo. The whole telencephalon of E14.5 embryos was dissected following the previously described method^33^. The dissected tissues were immediately placed in the tubes filled with the RNA*later*™ Stabilization Solution (Invitrogen) and stored at −80 °C by following the guidelines of the solution for long-term preservation. At the same time as the brain dissection, tail samples were collected from the embryos for sex determination using PCR (refer to ‘Sex determination’ section). Following sex determination, RNA was extracted from the telencephalon using the RNeasy Plus Mini Kit (QIAGEN). Sequencing of expression products (RNA-seq) for a total of 12 animals was commissioned by Takara Bio Inc. using samples from three male and three female telencephalons at E11.5 and E14.5, respectively. cDNA was synthesized with TruSeq Stranded mRNA Sample Prep Kit (Illumina) using XT-Auto System (Agilent). Subsequently, the generated libraries were then sequenced on HiSeq. 2500 platform (Illumina) with 100 bp paired end modules. The resulting sequenced reads were aligned to mouse reference genome GRCm38 (mm10) using STAR (v2.6.0c) and annotated using Genedata Proflier Genome (v13.0.11), which yielded mapped reads ranging from 46 to 75 million per sample.

### qRT-PCR

Samples were collected from the telencephalon of each embryo at E14.5. Total RNA was extracted using the RNeasy Mini Kit (QIAGEN) according to the manufacturer’s instructions. Subsequently, reverse transcription (RT) was carried out with the SuperScript™III First-Strand Synthesis System (Invitrogen). qRT-PCR was conducted as previously described^34^, in duplicate with TaqPro Universal SYBR qPCR Master Mix (Vazyme). The relative expression levels of the target genes were calculated using the 2^−ΔΔCt^ method, with *Sdha* serving as internal control. Primers were designed with Primer3Plus (https://www.bioinformatics.nl/cgi-bin/primer3plus/primer3plus.cgi) and listed in Supplementary Table S1. At least eight embryos of each sex were examined.

### Brain tissue sampling

To ensure consistent fluorescence immunostaining and fixation, perfusion fixation was performed on mouse embryos at E14.5 and E17.5. After removing the embryos from the pregnant mother, 4% paraformaldehyde (PFA, Sigma) dissolved in phosphate-buffered saline (PBS: 137 mM NaCl, 1.34 mM KCl, 10.1 mM Na2HPO4, 1.76 mM KH2PO4) was used for perfusion fixation. A borosilicate glass capillary (outer diameter: 1.5 mm, inner diameter: 0.86 mm) was attached to a peristaltic pump (ATTO). A 23-G needle (Terumo) was inserted through the left ventricle and then the right atrium was subsequently incised. Finally, 4% PFA was perfused at a flow rate of 3 mL per minute through the left ventricle of the embryo until no visible blood remained in the head^35^. The capillaries were prepared using a puller (PB-7, NARISHIGE), and the tips were sharpened with a forceps. Perfusion fixation was not performed on E11.5 mouse embryos.

For E11.5 mouse embryos, whole bodies were removed from pregnant mothers. For E14.5 and E17.5 mouse embryos, brain primordia were removed and then fixed overnight under 4 °C with 4% PFA while being gently shaken. The tissue was subsequently washed three times with PBS for 15 min at room temperature. Next it was immersed in 10% sucrose/PBS and 20% sucrose/PBS overnight at 4 °C, respectively. After these steps, the tissue was embedded in O. C. T Compound (Tissue-Tek, Sakura), gently frozen on dry ice, and sectioned. The sections were stored in a −80 °C deep freezer until further preparation.

### Fluorescence immunohistochemistry

Fluorescence immunohistochemistry was employed to examine the tissue architecture of the embryonic brain, following the method described in previous papers^36^ with necessary modifications. First, 14 µm thick frozen coronal sections of the embryonic brain was prepared using a cryostat (CM3050S, Leica). Sections taken at approximately 140 µm intervals for embryos at E11.5 and at approximately 200 µm intervals for embryonic brains at E14.5 and E17.5 were mounted on MAS-coated glass slides (Matsunami) and air-dried at room temperature for 15 minutes using a negative pressure device (Kartell). The prepared glass slides with the sections were stored in a −80 °C deep freezer until ready for use.

The sections were washed three times at room temperature for 5 minutes each in a staining bath containing 0.1% Triton X-100/TBS (TBST, Tris-buffered saline: 137 mM NaCl, 2.68 mM KCl, 24.8 mM Tris-base, pH 7.4). After the washes, excess water was gently drained, and a water-repellent line was drawn around the sections using a PAP Pen (Cosmo Bio). Next, a blocking solution composed of 3% bovine serum albumin (SIGMA) in 0.1% TBST was added onto the sections, and blocking was performed at room temperature for 30 minutes in a humidified chamber. The primary antibody solution was added to the sections and incubated overnight at 4 °C in a humidified chamber (see Supplementary Table S2 for the dilution ratios of the antibodies).

Following the primary antibody reaction, the sections were washed three times with TBST for 5 minutes each at room temperature in a staining glassware. Secondary antibody solution was added to the sections and incubated at room temperature in the dark for 1 hour (see Supplementary Table S2 for the dilution ratios of the antibodies). After completion of the secondary antibody reaction, the sections were washed three times with TBST for 5 minutes each at room temperature in a staining glassware. Excess water was removed, and the sections were sealed and stored using VECTASHIELD (VECTOR). Immunostaining images were captured using an all-in-one fluorescence microscope (BZ-X710, KEYENCE) and a confocal microscope (LSM800, Zeiss).

### *In situ* hybridization

*In situ* hybridization using frozen sections was performed as previously described^32,36^. The E14.5 WT telencephalon samples were used for RNA extraction using RNeasy Plus Mini Kit (QIAGEN). The extracted RNA was used for cDNA synthesis using the SuperScript™III First-Strand Synthesis System for RT-PCR (Invitrogen). The cDNA fragments of *Eif2s3x* (NM_012010, nucleotides 1–600), *Eif2s3y* (NM_012011, nucleotides 18–618), *Kdm6a* (NM_009483, nucleotides 1284–2005) or *Kdm5d* (NM_011419, nucleotides 1298–1897) were cloned into pBluescript II SK (–) (Stratagene). The RNA probes were synthesized using the cloned plasmids by the DIG RNA labeling kit (Roche). The antisense RNA probes for four genes were generated with T3 RNA polymerase (Promega). The sense RNA probes for *Eif2s3y* and *Kdm5d* were generated with T7 RNA polymerase (Promega) and were used to confirm the specificity of hybridization signals using antisense probes. For observation, sections were visualized with a microscopy system (BZ-X710, KEYENCE).

### Quantitative analysis of tissue architecture of the embryonic mouse cortical primordium

The histological structure of the cortical primordium of the mouse embryo was categorized into four regions: ventricular zone (VZ, positive for Pax6), subventricular zone (SVZ, Pax6 negative and Tbr2 positive), intermediate zone (IZ, Tuj1 positive nerve fibers), and cortical plate (CP, Tuj1 positive cell bodies)^37^. To analyze the area of the cortical primordium, a method described in a previous study was employed. A 200 µm wide segment was created at the midpoint of a straight line drawn from the dorsoventral border of the telencephalon to the dorsal and medial border of the mantle^38^. The area of the four brain regions within this segment was measured. The statistical test was carried out for both raw values and relative area of the four regions adjusted to total area of the cortex.Manual area measurements were conducted using the region of interest (ROI) manager tool of Fiji software. This approach allowed for the quantitative analysis of the tissue architecture of the cortical primordium in mouse embryo, providing valuable insights into its structural organization. The experimenter was not blinded to the sex of the samples.

### Statistics

All the statistics were performed using Python. The results of the statistical tests are summarized in “a dataset of results for statistical test.xlsx” in figshare^28^.

## Data Records

The following eight files related to RNA-seq and histological analysis data derived from male and female embryonic telencephalons have been deposited in figshare^28^.

a dataset of detailed descriptions of each file in figshare and GEO.docx

a dataset of expression_fpkm_counts.xlsx

a dataset of differentially expressed genes in the E14.5 telencephalons.xlsx

a dataset of gene list in Fig. 3A .xlsx

a dataset of HistologicalAnalysis.xlsx

a dataset of Immunohistochemistry_images.zip

a dataset of Immunohistochemistry_images_ScaleBar.zip

a dataset of Immunohistochemistry_images_analysis_ScaleBar.zip

a dataset of results for statistical test.xlsx

The following 24 files of raw RNA-seq data and one file of expression counts using RNA-seq FPKM derived from male and female embryonic telencephalons have been deposited in NCBI Gene Expression Omnibus (GEO)^29^.

24 raw RNA-seq files are named as follows e11/14 (E11 or E14, developmental stages) _male/female_1/2/3_R1/2.fastq. RNA-seq data are three replicates. The name of excel file is a dataset of expression_fpkm_counts.xlsx. “A dataset of detailed descriptions of each file in figshare and GEO.docx” is available in figshare for detailed descriptions and deposition website of each file.

## Technical Validation

### Sample information

Three embryos per each of four groups (E11.5 male, E11.5 female, E14.5 male, and E14.5 female) were used for the analyses of bulk RNA-seq; three male and three female embryos were selected from two litters for E11.5 groups. For E14.5 groups, two embryos were originated from the same litter and an additional one embryo came from another litter (Supplementary Table S3). For immunohistochemistry, we analyzed six groups including three male and female telencephalic samples at E11.5, ten male and female samples at E14.5, and four male and female samples at E17.5. We used more sample for E14.5 embryos since we expected there might be cytoarchitectural difference at this stage in consistent with the sex difference in gene expression profile at E14.5 (Fig. 4B,C, Supplementary Table S4). For E17.5 embryos where we found cytoarchitectural difference, we additionally quantified six sections per embryo with four samples to check the reproducibility of the results.

### RNA quality check

The quality of total RNA was assessed using the 2200 TapeStation (Agilent) by evaluating the RNA concentration, total amount, and the ratios of 260/280 and 260/230. All samples of RNA Integrity Number (RIN) values were 9.8 and more, which is sufficient to perform the RNA-seq analyses (Supplementary Table S3).

### Principal component analysis

To validate the data-collection procedure, inter-group comparisons were conducted between females and males. This involved performing a Principal Component Analysis (PCA) using the R programming language, specifically the “prcomp” command. Prior to the PCA, certain genes were deemed unreliable and thus excluded based on predefined criteria. These criteria included genes with zero variance of expression values within both male and female telencephalons groups or with a count value less than 16 in each group. As a result of PCA clustering, samples from same biological group were clustered closely (Fig. 1C) indicating validity of our RNA-seq data.

### Volcano plot

A volcano plot was generated using Python with the seaborn.scatterplot command. This plot visually represented the ratio of gene expression between males and female telencephalons (Log_2_(female expression (FPKM)/male expression)) on the horizontal axis and the confidence level based on statistical analysis (Log_10_(*q*-value)) on the vertical axis. Specific details regarding *q*-values can be found in the “Statistical Processing” section. Genes meeting the conditions of *q*-value < 0.05 and an absolute Log_2_(female expression level/male expression level) > 0.5 were identified as Differentially Expressed Genes (DEGs) in both males and females, and their count was determined. Most of the DEGs appeared with the criteria at E11.5 and a part of DEGs at E14.5 (Fig. 1D,E, Supplementary Table S5–9, ‘List of differentially expressed genes in the E14.5 telencephalons’ in figshare^28^) were consistent with prior studies utilizing microarray analysis and RT-PCR of the entire mouse brain at E10.5, E11.5 and E14.5^25–27^, indicating validity of our RNA-seq data^28,29^.

### *In situ* hybridization

*In situ* hybridization showed the reproducibility of the RNA-seq data. DEGs showing high fold change were selected: *Kdm6a* and *Eif2s3x* (female dominant expression), *Kdm5d* and *Eif2s3y* (male dominant expression). As a result, *Kdm6a* and *Eif2s3x* showed stronger signal in the telencephalon of the female samples, while *Kdm5d* and *Eif2s3y* showed stronger signal in the male samples, which is in consistent with RNA-seq data (Fig. 1F), indicating the reproducibility and validity of the RNA-seq data.

### Hierarchical clustering and Gene Ontology analysis

**Fig. 2:**
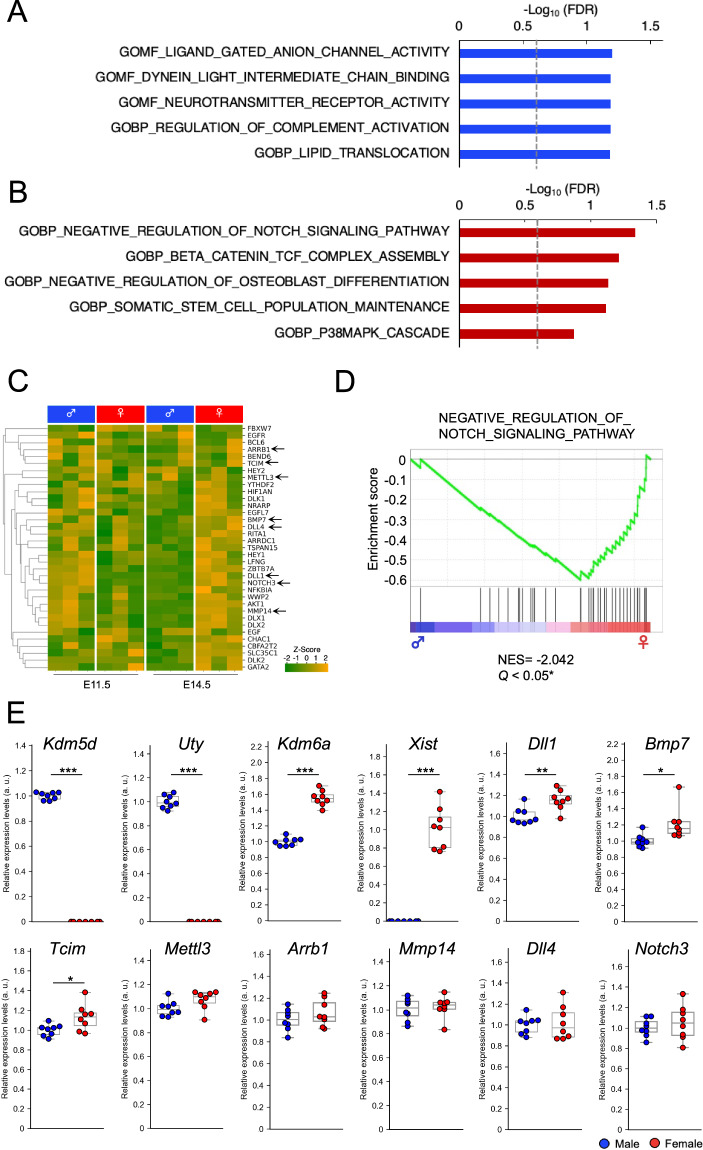
Gene Ontology analysis of differentially expressed genes between males and females at E14.5. (**A,B**) Gene Ontology (GO) analysis was performed on DEGs in male telencephalon (A, depicted in bule) or female telencephalons (B, decided in red) at E14.5. The horizontal axis represents the False Discovery Rate (FDR) on a logarithmic scale (-log_10_), and the dashed line indicates the statistically significant threshold with FDR < 0.25, corresponding to -Log_10_(FDR) > 0.60. Gene sets involved in the suppression of the Notch signaling pathway to maintain the undifferentiated state of neural stem cells (NSCs) are highly expressed in the female telencephalon. (**C**) A heatmap visualizes the gene expression in the GO category “NEGATIVE_REGULATION_OF_NOTCH_SIGNALING_PATHWAY.” The expression levels of many genes are similar between male and female telencephalons at E11.5. At E14.5, greater number of genes related to the Negative Regulation of Notch signaling pathway are highly expressed in the female telencephalon. The genes chosen for qRT-PCR validation are highlighted by arrows. (**D**) A Gene Set Enrichment Analysis (GSEA) plot depicts genes associated with the GO category “NEGATIVE_REGULATION_OF_NOTCH_SIGNALING_PATHWAY” at E14.5. The left side (blue) represents genes preferentially expressed in males, while the right side (red) represents genes preferentially expressed in females. The gene sets related to the aforementioned GO category are more abundant in the female telencephalon. (**E**) qRT-PCR analysis of the mRNA expression levels of the genes involved in “NEGATIVE_REGULATION_OF_NOTCH_SIGNALING_PATHWAY” and XY-linked genes in the telencephalon of male and female mice at E14.5 (n = 8). *Kdm6a*, *Xist*, *Dll1*, *Bmp7* and *Tcim* showed female dominant expression levels, while *Kdm5d* and *Uty* showed male dominant expression levels as in the case of RNA-seq data. Unpaired Student’s *t*-test *p < 0.05, **p < 0.005, ***p < 0.001.

**Fig. 3:**
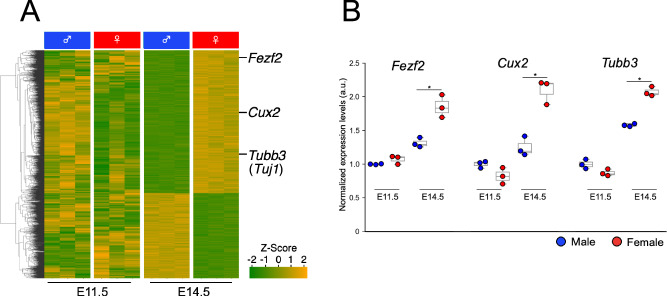
Neuronal genes are up-regulated in the female telencephalon at E14.5. (**A**) A heatmap illustrating the gene expression of the DEGs in the male and female telencephalon at E11.5 and E14.5. Neuronal genes, including *Fezf2*, *Cux2*, and *Tubb3* (Tuj1), show higher expression in the female telencephalons at E14.5. (**B**) Relative expression levels of *Fezf2*, *Cux2*, and *Tubb3* based on RNA-Seq data. The expression levels are normalized to that of the male telencephalon at E11.5, which is adjusted to a value of 1.0. *q < 0.05. The *q*-value represents the *p*-value from the Student’s *t*-test with multiple comparisons correction using the Benjamini-Hochberg method.

Hierarchical clustering and heatmap generation were performed using Python with the seaborn.clustermap command. These analyses aimed to characterize groups of DEGs in males and females. Additionally, a Gene Ontology (GO) gene sets from the Molecular Signatures Database (https://www.gsea-msigdb.org/gsea/msigdb/)^39^ was utilized to provide functional insights into these DEGs. Gene Set Enrichment Analysis (GSEA), as described in previous studies by^40–42^, was employed. As a result, genes associated with the suppression of the Notch signaling pathway displayed elevated expression levels in the female telencephalon at E14.5 (Fig. 2B–D, Supplementary Table S10). Notch signal is activated in the undifferentiated NSCs and inhibited in neurons and neural precursor cells^34,43^, and we found suppression of Notch signaling pathway as indicated by GSEA analysis (Fig. 3A,B). Consistently, *Tuj1* (*Tubb3*, a neuronal marker), *Fezf2* (a marker for layer V neurons), and *Cux2* (a marker for layer II-IV neurons) demonstrated higher expression levels in the female telencephalon at E14.5 (Fig. 3B). At E14.5, *Tuj1* is expressed in immature neurons^44^, and *Fezf2* and *Cux2* are expressed in early differentiated neurons^45,46^. These results support the consistency of our data.

### qRT-PCR

qRT-PCR was conducted to verify the reproducibility of the sex differences in the expression levels of the eight genes involved in GO term “NEGATIVE_REGULATION_OF_NOTCH_SIGNALING_PATHWAY”: *Dll1* (Delta like canonical Notch ligand 1), *Bmp7* (Bone morphogenetic protein 7), *Tcim* (transcriptional and immune response regulator), *Mettl3* (methyltransferase 3, N6-adenosine-methyltransferase complex catalytic subunit), *Arrb1* (Arrestin beta 1), *Mmp14* (Matrix metallopeptidase 14), *Dll4* (Delta like canonical Notch ligand 4) and *Notch3* (Notch receptor 3). Additional qRT-PCR assays were performed for *Kdm5d*, *Uty* (Y-linked genes), as well as *Kdm6a* and *Xist* (X-linked genes), to validate our qRT-PCR technique. These assays were conducted on new cohort of samples, with eight biological replicates for male and female at E14.5. The results confirmed clear sex difference in expression of *Kdm5d*, *Uty*, *Kdm6a* and *Xist* in consistent with RNA-seq data (Fig. 2E). Among the GO term, *Dll1*, *Bmp7* and *Tcim* showed higher expression in female than that of male, as in the case of the RNA-seq analysis (Fig. 2E). These findings support the reliability of our qRT-PCR data and RNA-seq data.

### Histological analysis

**Fig. 4:**
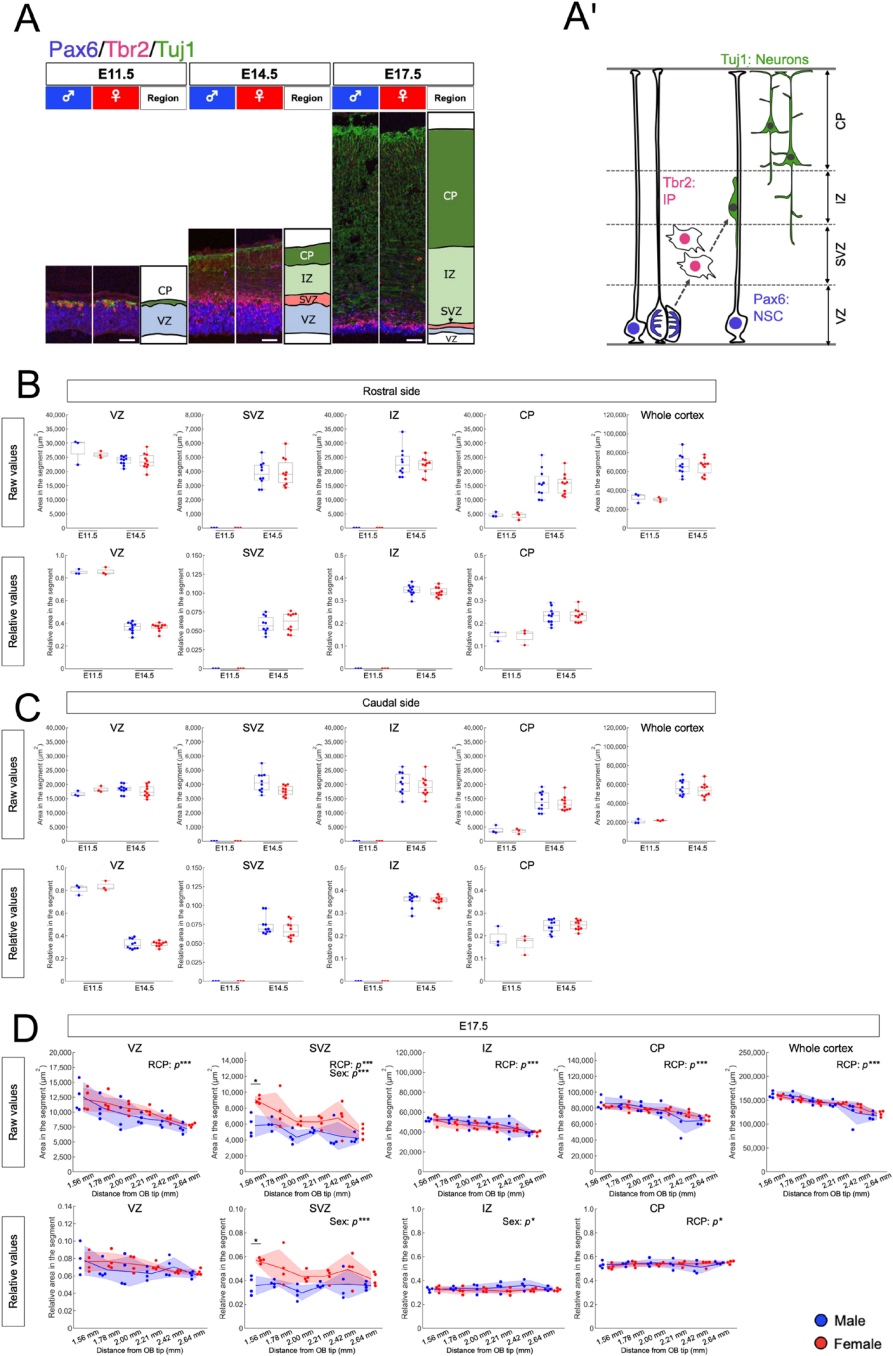
Analysis of sex differences in tissue organization in the embryonic mouse cerebral cortex primordium. (**A**) Tissue organization and schematic representation in the embryonic mouse cerebral cortex primordium at E11.5, 14.5, and 17.5. Immunohistochemistry was performed to measure the thickness of specific regions: ventricular zone (VZ, positive for Pax6), subventricular zone (SVZ, Pax6 negative and Tbr2 positive), intermediate zone (IZ, Tuj1 positive nerve fibers), and cortical plate (CP, Tuj1 positive cell bodies). Scale bar: 50 µm. (A’) Schematic representation of the different cell types involved in the construction of the embryonic mouse cerebral cortex primordium. The VZ contains the cell bodies of neural stem cells (NSCs), the SVZ contains intermediate progenitor cells (IPs), the IZ contains neuronal fibers and migrating immature neurons, and the CP contains a higher number of neurons. (B, C) Analysis of the raw area of VZ, SVZ, IZ, CP and the whole cortex within a 200 µm wide segment (upper panels) and the relative area of VZ, SVZ, IZ and CP compared to whole cortex (lower panel) at each developmental stage on the rostral (**B**) and caudal sides (**C**). (**D**) Analysis of the raw area of the VZ, SVZ, IZ, CP and the whole cortex within a 200 µm-wide segment (upper panels) and the relative area of the VZ, SVZ, IZ and CP compared to the whole cortex (lower panel) at E17.5 across six different rostro-caudal planes (1.56 mm to 2.64 mm from the tip of the olfactory bulb (OB)). Two-way ANOVA for rostro-caudal position (RCP)  × sex (*p < 0.05, ***p < 0.001), with post-hoc Tukey’s multiple comparison tests (*p < 0.05). The analysis includes three mice at E11.5, ten mice at E14.5 (**B,C**) and four mice at E17.5 (**D**) for both males and females.

We checked whether there are sex differences in the cytoarchitecture of the developing cortex by immunohistochemistry (Fig. 4A, also refer to the “Quantitative analysis of tissue architecture of the embryonic mouse cortical primordium” section for a detailed explanation of the defined neuroanatomical areas). As a result, there were no significant differences in the thickness of VZ, SVZ, IZ and CP between males and females at E11.5 and E14.5 (Fig. 4B,C), while the SVZ consistently showed greater thickness in the female telencephalon at E17.5 in both the raw values and the relative values normalized to the whole cortex size (Fig. 4D). This finding supports the exist of sex difference in gene expression levels at E14.5 that might result in the sex difference in the cytoarchitecture at the later stages. These experimental and analytical procedures were carried out to ensure the technical rigor and reliability of the dataset used in this study.

### Supplementary information

Supplementary Tables

## Data Availability

The open access software and versions mentioned in the main text were utilized for quality control and data analysis as described in the methods. 1. The University of California Santa Cruz (UCSC) Genome Browser was used to annotate mouse genetic information^47,48^: http://hgdownload.soe.ucsc.edu/goldenPath /mm10/database/ 2. The R command ‘prcomp’ was utilized for inter-group comparisons of PCA in male and female telencephalons. 3. The Python command ‘seaborn.scatterplot’ was utilized for generating a Volcano plot in males and female telencephalons. 4. The Python command ‘seaborn.clustermap’ was utilized for generating hierarchical clustering and heatmap. 5. The Gene Ontology (GO) gene set from the Molecular Signatures Database was utilized^39^: https://www.gsea-msigdb.org/gsea/msigdb/. All code and scripts used for data analysis in this study can be found in the following links and articles: http://hgdownload.soe.ucsc.edu/goldenPath /mm10/database/, https://www.r-project.org/help.html, https://www.gsea-msigdb.org/gsea/msigdb/^39,47,48^.
