# Supplementary material for: A Transcriptomic Dataset of Embryonic Murine Telencephalon
Source: Sci Data. 2024 Jun 5;11:586. doi: 10.1038/s41597-024-03421-x (PMC11153524; doi:10.1038/s41597-024-03421-x)
Supplement: Supplementary file 1 — Supplementary Tables [file 41597_2024_3421_MOESM1_ESM.pdf]

Supplementary Table S1. Primer sequences for qRT-PCR.

Gene	Forward primer	Reverse primer
Sdha	GCTCCTGCCTCTGTGGTTGA	AGCAACACCGATGAGCCTG
Bmp7	ATTGCACCTGAAGGCTATGC	TGGGTACTGTGTCTGGGTTG
Notch3	GAAAAATGGAGCCAACAAGG	GCAGCCTGTCCAAGTGATCT
Arrb1	GGAGCGACTCATCAAGAAGC	GCAGAGTGACTGAGCATGGA
Mmp14	CTGGGAAGGAATCCCTGAAT	ACTTGGGATACCCTGGCTCT
Dll1	TCAGATAACCCTGACGGAGGC	AGGTAAGAGTTGCCGAGGTCC
Dll4	TTGCTCTCCCAGGGACTCTA	TCTGAGTAGGCTCCTGCCTTA
Tcim	CGACCAAGATTTGGAGGAGA	CCATTGGTGGATACGCTCTT
Mettl3	CTGGGCACTTGGATTTAAGGAA	TGAGAGGTGGTGTAGCAACTT
Kdm5d	AGTTGTTCGTACAAACCAGTGT	CTGGCGTCCAACAGGTAGC
Uty	GAGGTTTTGTGGCATGGGAG	TGCAGAAGATAACGAAGGAGCTA
Kdm6a	CGGGCGGACAAAAGAAGAAC	CATAGACTTGCATCAGATCCTCC
Xist	GCCCAAAGGGACAAACAATC	GTAGCGAGGACTTGAAGAGAAG

Supplementary Table S2. List of antibodies for immunohistochemistry.

Antigen	Host	Manufacturers	Catalog	Dilution
Pax6	Rabbit	MBL	PD022	1:1000
Tbr2	Chicken	Millipore	AB15894	1:500
Tuj1	Mouse	BIoLegend	801201	1:2000
Anti-Chicken-IgG-Alexa488	Chicken	Jackson ImmunoResearch	AB_2337390	1:500
Anti-Rabbit-IgG-Cy3	Rabbit	Jackson ImmunoResearch	AB_2307443	1:500
Anti-Mouse-IgG-Alexa647	Mouse	Jackson ImmunoResearch	AB_2338902	1:500

Supplementary Table S3. Embryo and mother details for bulk RNA-seq at E11.5 and E14.5. Details on the embryos and their respective mothers for the bulk RNA-seq at E11.5 and E14.5. The table includes the following five key parameters: Sample ID, Sequence ID, Mother ID, the number of pups, and RNA Integrity Number (RIN).

	Sample ID	Sequence ID	Mother ID	# of pups	RIN
E11.5_male	R15-7	PR0657_04_a	R15	9	10.0
	R17-6	PR0657_05_a	R17	9	10.0
	R18-4	PR0657_06_a	R18	8	10.0
E11.5_female	R15-4	PR1336_04_a	R15	9	9.9
	R17-3	PR1336_05_a	R17	9	9.9
	R18-3	PR1336_06_a	R18	8	9.9
E14.5_male	R2-3	PR0721_04_a	R2	8	9.8
	R2-4	PR0721_05_a	R2	8	9.9
	R8-1	PR0721_06_a	R8	7	9.9
E14.5_female	R2-1	PR1190_04_a	R2	8	10.0
	R2-2	PR1190_05_a	R2	8	10.0
	R8-2	PR1190_06_a	R8	7	10.0

Supplementary Table S4. Litter information for immunohistochemistry at E11.5, E14.5 and E17.5.

Sample serial number	Mother ID	Embryo ID	Stage	Sex	Rostro-caudal position	Image ID
1	592	4	E11.5	Male	Rostral_A	1_E11_Male1_Rostral_A
1	592	4	E11.5	Male	Caudal_A	2_E11_Male1_Caudal_A
2	592	5	E11.5	Male	Rostral_A	3_E11_Male2_Rostral_A
2	592	5	E11.5	Male	Caudal_A	4_E11_Male2_Caudal_A
3	592	6	E11.5	Male	Rostral_A	5_E11_Male3_Rostral_A
3	592	6	E11.5	Male	Caudal_A	6_E11_Male3_Caudal_A
4	592	1	E11.5	Female	Rostral_A	7_E11_Female4_Rostral_A
4	592	1	E11.5	Female	Caudal_A	8_E11_Female4_Caudal_A
5	592	2	E11.5	Female	Rostral_A	9_E11_Female5_Rostral_A
5	592	2	E11.5	Female	Caudal_A	10_E11_Female5_Caudal_A
6	592	3	E11.5	Female	Rostral_A	11_E11_Female6_Rostral_A
6	592	3	E11.5	Female	Caudal_A	12_E11_Female6_Caudal_A
7	528	3	E14.5	Male	Rostral_A	13_E14_Male7_Rostral_A
7	528	3	E14.5	Male	Caudal_A	14_E14_Male7_Caudal_A
8	528	4	E14.5	Male	Rostral_A	15_E14_Male8_Rostral_A
8	528	4	E14.5	Male	Caudal_A	16_E14_Male8_Caudal_A
9	528	6	E14.5	Male	Rostral_A	17_E14_Male9_Rostral_A
9	528	6	E14.5	Male	Caudal_A	18_E14_Male9_Caudal_A
10	570	2	E14.5	Male	Rostral_A	19_E14_Male10_Rostral_A
10	570	2	E14.5	Male	Caudal_A	20_E14_Male10_Caudal_A
11	570	4	E14.5	Male	Rostral_A	21_E14_Male11_Rostral_A
11	570	4	E14.5	Male	Caudal_A	22_E14_Male11_Caudal_A
12	570	5	E14.5	Male	Rostral_A	23_E14_Male12_Rostral_A
12	570	5	E14.5	Male	Caudal_A	24_E14_Male12_Caudal_A
13	683	10	E14.5	Male	Rostral_A	25_E14_Male13_Rostral_A
13	683	10	E14.5	Male	Caudal_A	26_E14_Male13_Caudal_A
14	745	2	E14.5	Male	Rostral_A	27_E14_Male14_Rostral_A
14	745	2	E14.5	Male	Caudal_A	28_E14_Male14_Caudal_A
15	745	4	E14.5	Male	Rostral_A	29_E14_Male15_Rostral_A
15	745	4	E14.5	Male	Caudal_A	30_E14_Male15_Caudal_A
16	745	8	E14.5	Male	Rostral_A	31_E14_Male16_Rostral_A
16	745	8	E14.5	Male	Caudal_A	32_E14_Male16_Caudal_A
17	528	5	E14.5	Female	Rostral_A	33_E14_Female17_Rostral_A
17	528	5	E14.5	Female	Caudal_A	34_E14_Female17_Caudal_A
18	528	7	E14.5	Female	Rostral_A	35_E14_Female18_Rostral_A
18	528	7	E14.5	Female	Caudal_A	36_E14_Female18_Caudal_A
19	528	8	E14.5	Female	Rostral_A	37_E14_Female19_Rostral_A
19	528	8	E14.5	Female	Caudal_A	38_E14_Female19_Caudal_A
20	570	1	E14.5	Female	Rostral_A	39_E14_Female20_Rostral_A
20	570	1	E14.5	Female	Caudal_A	40_E14_Female20_Caudal_A
21	570	7	E14.5	Female	Rostral_A	41_E14_Female21_Rostral_A
21	570	7	E14.5	Female	Caudal_A	42_E14_Female21_Caudal_A
22	570	9	E14.5	Female	Rostral_A	43_E14_Female22_Rostral_A
22	570	9	E14.5	Female	Caudal_A	44_E14_Female22_Caudal_A
23	683	1	E14.5	Female	Rostral_A	45_E14_Female23_Rostral_A
23	683	1	E14.5	Female	Caudal_A	46_E14_Female23_Caudal_A
24	683	4	E14.5	Female	Rostral_A	47_E14_Female24_Rostral_A
24	683	4	E14.5	Female	Caudal_A	48_E14_Female24_Caudal_A
25	683	5	E14.5	Female	Rostral_A	49_E14_Female25_Rostral_A
25	683	5	E14.5	Female	Caudal_A	50_E14_Female25_Caudal_A
26	745	5	E14.5	Female	Rostral_A	51_E14_Female26_Rostral_A
26	745	5	E14.5	Female	Caudal_A	52_E14_Female26_Caudal_A
27	100	1	E17.5	Male	Rostral_A	53_E17_Male27_Rostral_A
27	100	1	E17.5	Male	Rostral_B	54_E17_Male27_Rostral_B
27	100	1	E17.5	Male	Rostral_C	55_E17_Male27_Rostral_C
27	100	1	E17.5	Male	Caudal_A	56_E17_Male27_Caudal_A
27	100	1	E17.5	Male	Caudal_B	57_E17_Male27_Caudal_B
27	100	1	E17.5	Male	Caudal_C	58_E17_Male27_Caudal_C
28	112	1	E17.5	Male	Rostral_A	59_E17_Male28_Rostral_A
28	112	1	E17.5	Male	Rostral_B	60_E17_Male28_Rostral_B
28	112	1	E17.5	Male	Rostral_C	61_E17_Male28_Rostral_C
28	112	1	E17.5	Male	Caudal_A	62_E17_Male28_Caudal_A

28	112	1	E17.5	Male	Caudal_B	63_E17_Male28_Caudal_B
28	112	1	E17.5	Male	Caudal_C	64_E17_Male28_Caudal_C
29	94	8	E17.5	Male	Rostral_A	65_E17_Male29_Rostral_A
29	94	8	E17.5	Male	Rostral_B	66_E17_Male29_Rostral_B
29	94	8	E17.5	Male	Rostral_C	67_E17_Male29_Rostral_C
29	94	8	E17.5	Male	Caudal_A	68_E17_Male29_Caudal_A
29	94	8	E17.5	Male	Caudal_B	69_E17_Male29_Caudal_B
29	94	8	E17.5	Male	Caudal_C	70_E17_Male29_Caudal_C
30	93	1	E17.5	Male	Rostral_A	71_E17_Male30_Rostral_A
30	93	1	E17.5	Male	Rostral_B	72_E17_Male30_Rostral_B
30	93	1	E17.5	Male	Rostral_C	73_E17_Male30_Rostral_C
30	93	1	E17.5	Male	Caudal_A	74_E17_Male30_Caudal_A
30	93	1	E17.5	Male	Caudal_B	75_E17_Male30_Caudal_B
30	93	1	E17.5	Male	Caudal_C	76_E17_Male30_Caudal_C
31	100	4	E17.5	Female	Rostral_A	77_E17_Female31_Rostral_A
31	100	4	E17.5	Female	Rostral_B	78_E17_Female31_Rostral_B
31	100	4	E17.5	Female	Rostral_C	79_E17_Female31_Rostral_C
31	100	4	E17.5	Female	Caudal_A	80_E17_Female31_Caudal_A
31	100	4	E17.5	Female	Caudal_B	81_E17_Female31_Caudal_B
31	100	4	E17.5	Female	Caudal_C	82_E17_Female31_Caudal_C
32	112	3	E17.5	Female	Rostral_A	83_E17_Female32_Rostral_A
32	112	3	E17.5	Female	Rostral_B	84_E17_Female32_Rostral_B
32	112	3	E17.5	Female	Rostral_C	85_E17_Female32_Rostral_C
32	112	3	E17.5	Female	Caudal_A	86_E17_Female32_Caudal_A
32	112	3	E17.5	Female	Caudal_B	87_E17_Female32_Caudal_B
32	112	3	E17.5	Female	Caudal_C	88_E17_Female32_Caudal_C
33	94	4	E17.5	Female	Rostral_A	89_E17_Female33_Rostral_A
33	94	4	E17.5	Female	Rostral_B	90_E17_Female33_Rostral_B
33	94	4	E17.5	Female	Rostral_C	91_E17_Female33_Rostral_C
33	94	4	E17.5	Female	Caudal_A	92_E17_Female33_Caudal_A
33	94	4	E17.5	Female	Caudal_B	93_E17_Female33_Caudal_B
33	94	4	E17.5	Female	Caudal_C	94_E17_Female33_Caudal_C
34	93	3	E17.5	Female	Rostral_A	95_E17_Female34_Rostral_A
34	93	3	E17.5	Female	Rostral_B	96_E17_Female34_Rostral_B
34	93	3	E17.5	Female	Rostral_C	97_E17_Female34_Rostral_C
34	93	3	E17.5	Female	Caudal_A	98_E17_Female34_Caudal_A
34	93	3	E17.5	Female	Caudal_B	99_E17_Female34_Caudal_B
34	93	3	E17.5	Female	Caudal_C	100_E17_Female34_Caudal_C

Supplementary Table S5. DEGs showing male bias in E11.5 bulk RNA-seq. Fold change in expression calculated as Log_2 (female expression / male expression). A positive fold change value indicates female-biased expression, while a negative value indicates male-biased expression. *q-value < 0.05 and $|Log_2$ (female expression / male expression) $| > 0.5$.

Gene Name	Chromosome	Fold Change
Eif2s3y	chrY	-17.3*
Kdm5d	chrY	-17.0*
Ddx3y	chrY	-17.0*
Uty	chrY	-16.0*
Scand1	chr2	-1.4*

Supplementary Table S6. DEGs showing female bias in E11.5 bulk RNA-seq. Fold change in expression calculated as Log_2 (female expression / male expression). A positive fold change value indicates female-biased expression, while a negative value indicates male-biased expression. *q-value < 0.05 and $|Log_2$ (female expression / male expression) $| > 0.5$.

Gene Name	Chromosome	Fold Change
Xist	chrX	13.9*
Kdm6a	chrX	0.6*
Eif2s3x	chrX	0.6*

Supplementary Table S7. Top 20 genes with male-dominated expression in E14.5 bulk RNA-seq. The fold change in expression calculated as Log_2 (female expression / male expression). A positive fold change value indicates female-biased expression, while a negative value indicates male-biased expression. **q*-value < 0.05 and $|Log_2$ (female expression / male expression) $| > 0.5$.

Gene Name	Chromosome	Fold Change
Eif2s3y	chrY	-18.0*
Kdm5d	chrY	-17.2*
Ddx3y	chrY	-17.2*
Uty	chrY	-16.1*
Btg3	chr16	-15.9*
Cebpb	chr2	-2.6*
Gm14853	chrX	-1.9*
Cd46	chr1	-1.8*
Gm15246	chrX	-1.4*
Gm30223	chr3	-1.4*
Ttc14	chr3	-1.4*
A230057D06Rik	chr7	-1.3*
C030023E24Rik	chrX	-1.3*
E330033B04Rik	chr15	-1.2*
Gm15328	chr18	-1.2*
OTTMUSG00000016609	chr2	-1.2*
Vmn2r1	chr3	-1.2*
Dnm3os	chr1	-1.2*
Cfap47	chrX	-1.2*
lpw	chr7	-1.2*

Supplementary Table S8. Top 20 genes with female-dominated expression in E14.5 bulk RNA-seq. The fold change in expression is calculated as Log_2 (female expression / male expression). A positive fold change value indicates female-biased expression, while a negative value indicates male-biased expression. **q*-value < 0.05 and |Log_2 (female expression / male expression) | > 0.5.

Gene Name	Chromosome	Fold Change
Xist	chrX	12.8*
Rpph1	chr14	5.2*
Rmrp	chr4	3.8*
Lars2	chr9	2.6*
Rn45s	chr17	2.5*
Xntrpc	chr7	1.5*
Hist1h2af	chr13	1.5*
Naprt	chr15	1.2*
Erdr1	chrX	1.1*
Mbd6	chr10	1.1*
Tfeb	chr17	1.1*
Kcp	chr6	1.0*
Col11a2	chr17	1.0*
Rpl37rt	chr5	1.0*
Ky	chr9	1.0*
Mlxipl	chr5	1.0*
Raver1	chr9	1.0*
Kfkbie	chr17	1.0*
Anxa11	chr14	1.0*
Nckap5l	chr15	0.9*

Supplementary Table S9. Genes on the Y chromosome and their corresponding homologous genes on the X chromosome identified in E11.5 bulk RNA-seq data. The fold change in expression is calculated as Log_2 (female expression / male expression). *$q < 0.05$ and $|Log_2$ (female expression / male expression)$| > 0.5$.

Genes on the chrY	Fold Change	Genes on the chrX	Fold Change
Elf2s3y	-17.3*	*Elf2s3x*	0.6*
Kdm5d<br>*(SMCY)*	-17.0*	*Kdm5c*<br>*(SMCX)*	0.3
Ddx3y<br>*(Ddy)*	-17.0*	*Ddx3x*<br>*(Ddx)*	0.0
Uty<br>*(Kdm6c)*	-16.0*	*Kdm6a*<br>*(Utx)*	0.6*

Supplementary Table S10. List of genes involved in the GO term "NEGATIVE_REGULATION_OF_NOTCH_SIGNALING_PATHWAY" with their location on the chromosomes and fold changes observed in E14.5 bulk RNA-seq. The fold change in expression is calculated as Log_2 (female expression / male expression). *$q < 0.05$ and $|Log_2$ (female expression / male expression) $| > 0.5$.

Gene Name	Chromosome	Fold Change
Dlk1	Chr12	0.8
Arrdc1	Chr2	0.7*
Dlx2	Chr2	0.7*
Dlk2	Chr17	0.7*
Notch3	chr17	0.7*
Gata2	Chr6	0.6*
Zbtb7a	chr10	0.6*
Akt1	chr12	0.5*
Rita1	Chr5	0.5*
Dll1	chr17	0.5
Chac1	Chr2	0.4
Mmp14	Chr14	0.4
Tspan15	chr10	0.4
Egfl7	Chr2	0.3
Hey1	Chr3	0.3
Slc35c1	Chr2	0.3
Lfng	Chr5	0.3
Dlx1	Chr2	0.3
Bmp7	Chr2	0.3
Nrarp	Chr2	0.3
Wwp2	Chr8	0.3
Dll4	Chr2	0.2
Nfkbia	Chr12	0.2
Arrb1	Chr7	0.1
Ythdf2	Chr4	0.1
Hif1an	Chr19	0.1
Tcim	Chr8	0.1
Cbta2t2	Chr2	0.0
Mettl3	Chr14	0.0
Egf	Chr3	0.0
Fbxw7	Chr3	-0.1
Bcl6	Chr16	-0.1
Hey2	Chr10	-0.1
Bend6	Chr1	-0.2
Egfr	Chr11	-0.6
